# Fluorinated Polyimide/Allomelanin Nanocomposites for UV-Shielding Applications

**DOI:** 10.3390/molecules28145523

**Published:** 2023-07-19

**Authors:** Qing Li, Yujuan Guo, Meijia Wu, Fei Deng, Jieying Feng, Jiafeng Liu, Sheng Liu, Chaoliu Ouyang, Wengui Duan, Shunmin Yi, Guangfu Liao

**Affiliations:** 1 School of Chemistry and Chemical Engineering, Guangxi University, Nanning 530004, China; liqing@hubu.edu.cn; 2 Ministry of Education Key Laboratory for the Green Preparation and Application of Functional Materials, Hubei Key Laboratory of Polymer Materials, School of Materials Science and Engineering, Hubei University, Wuhan 430062, China; yjguohubu@163.com (Y.G.); liushenggxun@163.com (S.L.); 3 Guangxi Colleges and Universities Key Laboratory of Environmental-friendly Materials and New Technology for Carbon Neutralization, Guangxi Key Laboratory of Advanced Structural Materials and Carbon Neutralization, School of Materials and Environment, Guangxi Minzu University, Nanning 530105, China; wmj19961177925@163.com (M.W.); d1409730272@163.com (F.D.); jieyingfeng1031@163.com (J.F.); 18207707530@163.com (J.L.); ouyang473033963@163.com (C.O.); shunminyi10@163.com (S.Y.); 4 College of Materials Engineering, Fujian Agriculture and Forestry University, Fuzhou 350002, China

**Keywords:** polyimide, allomelanin, mechanical properties, UV-shielding properties, nanocomposite

## Abstract

A series of highly fluorinated polyimide/allomelanin nanoparticles (FPI/AMNPs) films were prepared with FPI as the matrix and AMNPs as the filler. Due to the formation of hydrogen bonds, significantly reinforced mechanical and UV-shielding properties are acquired. Stress–strain curves demonstrated a maximum tensile strength of 150.59 MPa and a fracture elongation of 1.40% (0.7 wt.% AMNPs), respectively, 1.78 and 1.56× that of pure FPI. The measurements of the UV-vis spectrum, photodegradation of curcumin and repeated running tests confirmed the splendid UV-shielding capabilities of FPI/AMNPs films. The enhancement mechanisms, such as synergistic UV absorption of the charge transfer complexes in FPI and AMNPs and photothermal conversion, were the reasons for its exceptional UV shielding. The excellent comprehensive properties above enable FPI/AMNPs nanocomposites to be potential candidates in the field of UV shielding.

## 1. Introduction

Ultraviolet (UV) is a general term for radiation with a frequency of 750 THz~30 PHz in the electromagnetic spectrum, corresponding to a wavelength of 400~10 nm in a vacuum, and it can cause damage to people’s vision. It is invisible light with a higher frequency than blue-violet light. It can be divided into UVA (400~320 nm, low frequency long wave), UVB (320~280 nm, medium frequency medium wave), UVC (280~100 nm, high-frequency short wave) and EUV (100~10 nm, ultra-high frequency). Among them, UVA rays cause tanning, and UVB rays have shorter wavelengths that can burn the skin. UVC is normally blocked by the ozone layer. However, in space, without the protection of the ozone layer, the intensity of ultraviolet radiation increases sharply, especially high-energy UVC and EUV, damaging human health, accelerating the degradation of visual polymer fittings in high-altitude aircraft, aerospace spacecraft and satellites and so on, as well as causing damage to objects such as space caps, glass windows and packaging materials for a national flag or an organization’s logo. High-performance UV-shielding materials need to be developed urgently, especially for high-energy UVC and EUV shielding, which will promote the development of the aerospace field.

The commonly used scheme is to add UV absorbents to polymer substrates, including inorganic types, such as TiO_2_, SiO_2_, ZnO, CdS, etc. [1,2,3,4,5,6,7]. The special energy band structure endows them with an excellent UV absorption capacity and a high transmittance. However, owing to their catalytic capabilities, polymer substrates will degrade faster when exposed to light, decreasing the useful life of the composite materials [8,9,10,11]. Without degrading polymer substrates, 2D nanomaterials such as boron nitride and graphene oxide (GO) were reported as UV absorbents. However, easy agglomeration, poor compatibility, complex preparation process and high cost limited their UV-shielding applications [12,13,14,15,16,17,18,19,20,21]. Also, POSS can improve the UV-shielding properties of polymers through light scattering, but it also reduces their visible light transmittance [22,23,24,25]. Additionally, natural materials like melanin, esculetin, lignin, etc., can also be a useful option for UV absorption, especially for melanin, but their future utilization was found to be hindered by the difficult purifying procedure and volume production [26,27,28]. In our prior works [29], amino-modified melanin (ASE) and polydopamine (PDA) were prepared as fillers in nanocomposites to improve UV-shielding characteristics. However, the difficult mass production of ASE and the insufficient absorption of high-energy UVC by ASE and PDA limited their further use as UV absorbers.

Allomelanin, a type of melanin without nitrogen element, is composed of catechol and 1,8-dihydroxynaphthalene (1,8-DHN) [30,31,32,33,34]. Research shows that allomelanin can avoid damage from high doses of radiation, and fungi containing allomelanin can not only survive in radioactive environments, such as spaceships, nuclear power plants and Chernobyl reactors, but also convert the radiation into chemical energy needed for their own growth [33,35,36]. These serve to further highlight the broad range of potential applications for allomelanin in the high-energy ultraviolet-shielding field. To scale up the production of allomelanin, artificial allomelanin can be synthesized via oxidative oligomerization of 1,8-DHN in aqueous solution at room temperature [32], which solved the problem of the large-scale production of this kind of melanin.

Polyimide (PI) possesses outstanding comprehensive properties, such as resistance to high and low temperatures, excellent mechanical properties, dielectric properties and UV-shielding properties, and is regarded as one of the most promising high-performance engineering polymers in the 21st century [37,38,39,40]. At present, it is favored in the fields of PI fibers, insulation materials, aerospace, microelectronics and other fields, and it plays an important role in practical applications with different forms [8,41,42,43,44,45,46,47,48]. The main chain of PI contains electron donors (aromatic amine monomers) and electron acceptors (aromatic dianhydride monomers), and the alternating composition of the two groups leads to the formation of a large number of intramolecular and intermolecular charge transfer complexes (CTC), which endows the material certain UV-trapping capability [49]. This is especially the case for UVA and UVB, but not for high-energy UVC. Therefore, it is a feasible strategy to combine the appropriate UV absorber, allomelanin, with PI to achieve the synergistic absorption of UV radiation. It is worth noting that most studies on PI-based UV-shielding materials are usually doped with excessive nano-fillers [50,51]. This will not only bring about the increased cost of the materials, but it will also damage the mechanical properties of the material to a certain extent. Thus, it is necessary to reduce the amounts of nano-fillers to achieve the same or better UV-shielding performance.

Herein, a highly fluorinated polyimide with -CF_3_ was selected as the polymer matrix to successfully relieve the chrominance of PI [52,53], and AMNPs were added as fillers. Based on thermal imidization and the formation of hydrogen bonding between AMNPs and FPI, a group of FPI/AMNPs films with varying levels of AMNPs were obtained. The larger steric hindrance caused by fluorinated groups raised the porosity and depressed the density of FPI, which further broadened the propagation path of UV. Thus, UV-shielding performance was enhanced. So as to explore the effect of AMNPs supplemental level on the various performance of FPI, the structure of FPI/AMNPs films was characterized by FTIR, which confirmed the successful synthesis of the materials. The best mechanical properties and tensile mechanism of the films were determined via tensile test and sectional FESEM. In addition, the UV-shielding experiments also showed the enhancement of UV-shielding performance, confirming the synergistic absorption of UV by FPI and AMNPs. The main purpose of this work was to prepare high-performance FPI composite UV-shielding materials and to study the enhancement mechanism of AMNPs on the mechanical and UV-shielding properties of FPI. These results will be powerful evidence to promote the UV-shielding performance of aerospace materials.

## 2. Results and Discussion

### 2.1. Preparation and Characterization of AMNPs

**Figure 1:**
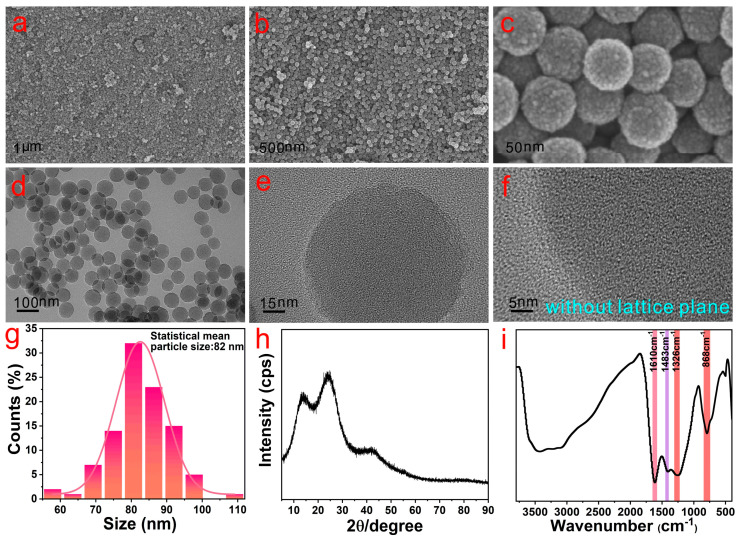
FESEM (**a**–**c**), TEM (**d**,**e**), high-resolution TEM image (**f**), XRD pattern (**h**) and FTIR spectrum (**i**) of AMNPs, respectively. The statistical size histograms and fitting curve (**g**) of (**d**).

AMNPs were synthesized via oxidative oligomerization of 1,8-DHN in aqueous solution at room temperature. It can be seen from FESEM and TEM images that AMNPs present a uniform spherical shape (Figure 1a–d) with relatively rough profile (Figure 1e). Moreover, no lattice planes are found in the high-resolution TEM image (Figure 1f), indicating that the structure was amorphous. According to the statistics in Figure 1d, AMNPs are in the range 57–108 nm with an average particle size of 82 nm (Figure 1g). In the XRD pattern (Figure 1h), three broad and blunt diffraction peaks successively appear at 14°, 25° and 42°, further verifying the amorphous structure of AMNPs. The FTIR spectrum of AMNPs is shown in Figure 1i. The sharp peaks at 3120 cm^−1^, 1610 cm^−1^, 1483 cm^−1^, 1326 cm^−1^ and 868 cm^−1^ correspond to aromatic C-H stretching, aromatic C=C stretching, C-OH bending, C-OH stretching and aromatic C-H bending, respectively. The broad peaks in the 3200–3400 cm^−1^ range are attributed to the stretching of -OH groups on the naphthalene ring. The FTIR spectrum also demonstrated the successful synthesis of AMNPs.

**Figure 2:**
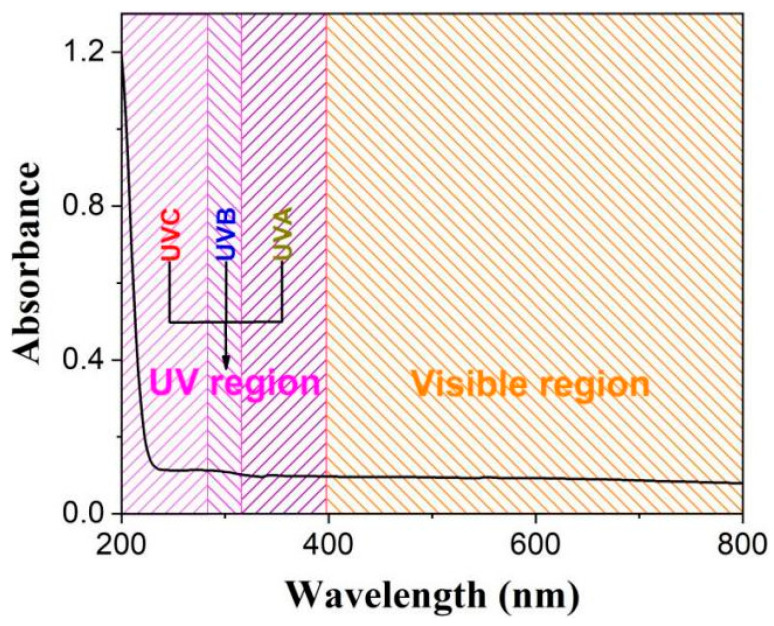
UV-vis absorption spectrum of AMNPs.

Figure 2 shows that AMNPs exhibit superior UV absorption, especially for high-energy UVC, and basically no visible light absorption, which shows they are a truly excellent high-energy ultraviolet absorber.

### 2.2. Analysis of FPI/AMNPs Films

For the sake of achieving UV-shielding films with high visible light transmittance based on PI, highly fluorinated PI was used as the substrate and AMNPs as the filler. The abundant and strongly electronegative trifluoromethyl inhibits the generation of intramolecular CT complexes and therefore results in a high transmittance in the visible light range [54]. Highly fluorinated PI can effectively enhance the transparency of the composites. The rough AMNPs possess favorable contact performance with FPI via hydrogen bonding linkages, so FPI/AMNPs nanocomposites with exceptional compatibility are prepared. To strengthen the films, thermal imidization was adopted to accomplish further treatment. As a result, the whole strategy of preparation is straightforward and efficient.

### 2.3. Structure Analysis

**Figure 3:**
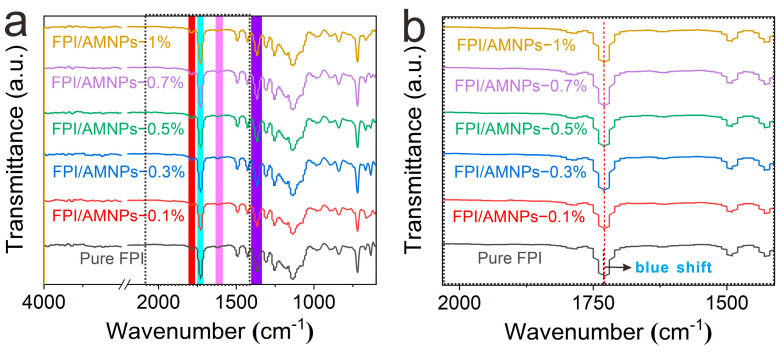
FTIR spectra of FPI/AMNPs films. (**b**) is an enlarged view of the dotted box area of (**a**).

The FTIR spectra were used to examine the changes in functional groups before and after the addition of AMNPs. There are distinctive imide peaks in all curves (Figure 3), which are ascribed to the asymmetric (1778 cm^−1^) and symmetric (1722 cm^−1^) stretching vibrations of C=O. The characteristic peak at 1377 cm^−1^ is derived from the stretching vibration of C-N. In addition, no characteristic peak is detected at 1660 cm^−1^, indicating that the amide was completely converted into imide. Additionally, the infrared curves of FPI and FPI/AMNPs films are strikingly similar, which is attributed to the existence of the same functional groups of FPI and AMNPs. Furthermore, we can see from Figure 3b that after adding AMNPs, the peak at 1722 cm^−1^ has shifted slightly to the low-frequency direction, which is the so-called blue shift. This is mainly due to the formation of hydrogen bonds between the carbonyl group of FPI and the hydroxyl group of AMNPs, which limits the stretching vibration of the carbonyl group. However, the degree of blue shift is small or even negligible. There are two reasons for this: the amount of AMNPs added is too small, resulting in limited hydrogen bonds, and the total reflection infrared test can only detect the hydrogen bonds on the surface. Therefore, the network structure of hydrogen bonding is effectively demonstrated.

### 2.4. Mechanical Properties

**Figure 4:**
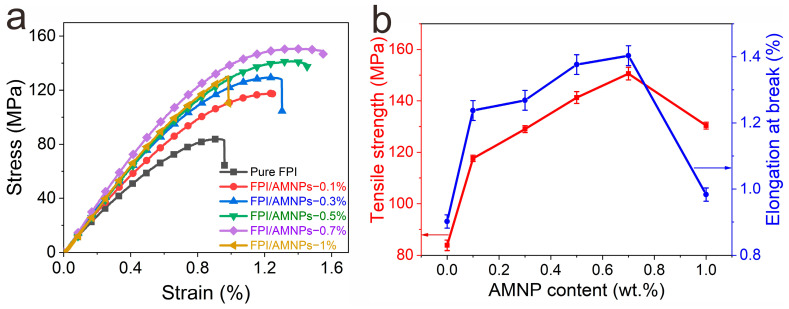
(**a**) Stress–strain curves of FPI/AMNPs films; (**b**) influences of AMNPs content on FPI/AMNPs films’ elongation and tensile strength at rupture.

Excellent mechanical strength is the basic premise of materials. Figure 4a shows that all films have brittle fracture behavior without an obvious yield point. Table 1 summarizes the average tensile strength (σ) and elongation at break (ε_max_) of the films. With the increase in AMNPs content, σ and ε_max_ first increase and then decrease, reaching the maximum values at 0.7 wt.%, which are 1.78 and 1.56 times of the initial FPI, respectively (Figure 4b). Furthermore, in the optimal formulation, σ (150.59 MPa) is a much greater value than that of our previous works [8,9], and the amount of filler is also at a lower level (0.7 wt.%), greatly reducing the cost of raw materials.

**Figure 5:**
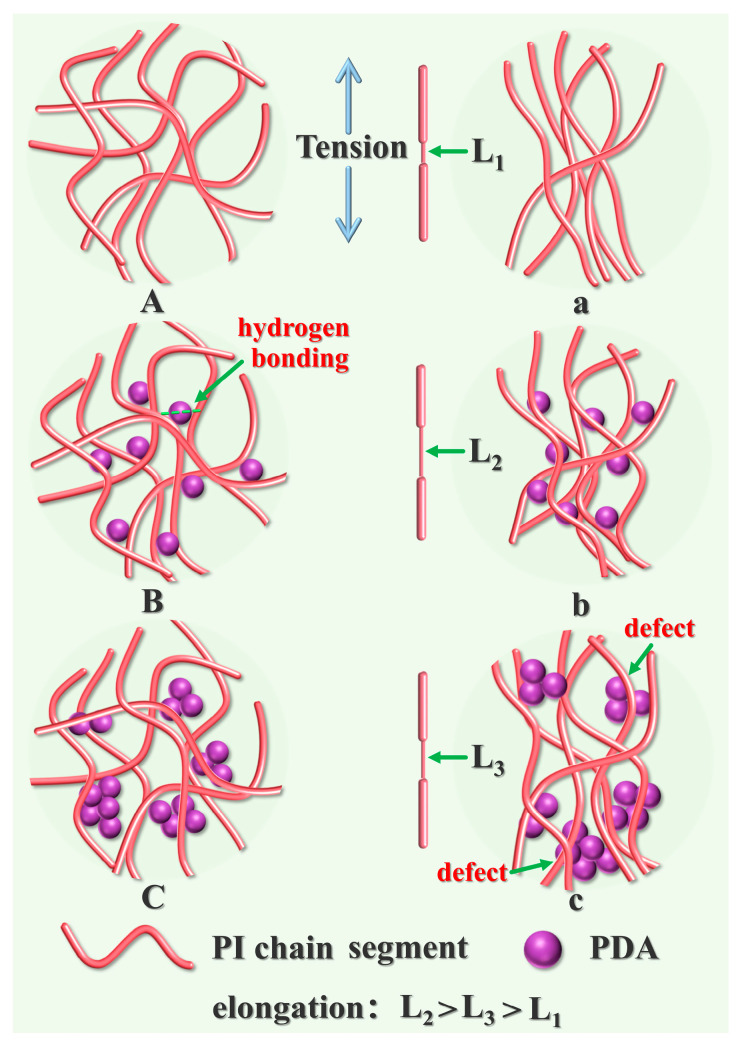
The conceptual pictures of AMNPs dispersed in FPI matrix: (**A**) pure FPI, (**B**) well-dispersed FPI/AMNPs networks at low AMNPs loading, (**C**) AMNPs aggregate in FPI matrix at high loading. (**a**–**c**) are pictures after tension, respectively.

The connection between the particle system and the interaction between nanoparticles and polymers is one of the key factors regulating the mechanical properties of films [55,56,57]. The tensile mechanism is shown in Figure 5. The molecular chains of pure FPI contained only random physical entanglement, which rapidly oriented and produced a fracture when subjected to stress (Figure 5(A,a)). With the addition of AMNPs, a large number of hydrogen bonds were formed between AMNPs and FPI, which resulted in the transformation of the topological structure of FPI molecular chains from physical entanglement to hydrogen bond crosslinking entanglement. The scattered AMNPs acted as the connection points (Figure 5(B,b)), slowing down the stress concentration in the structure. When the stress increased further, the FPI chains’ slip was strengthened. A high orientation was obtained to withstand more stress. As a result, both σ and ε_max_ increased to the maximum. Meanwhile, the sliding friction caused by FPI led to a hysteresis loss, by which part of the eliminated external force is further converted into heat. However, excessive AMNPs aggregated in the FPI matrix because of the limited carbonyl group, which induced the generation of defects and stress concentration (Figure 5(C,c)).

**Figure 6:**
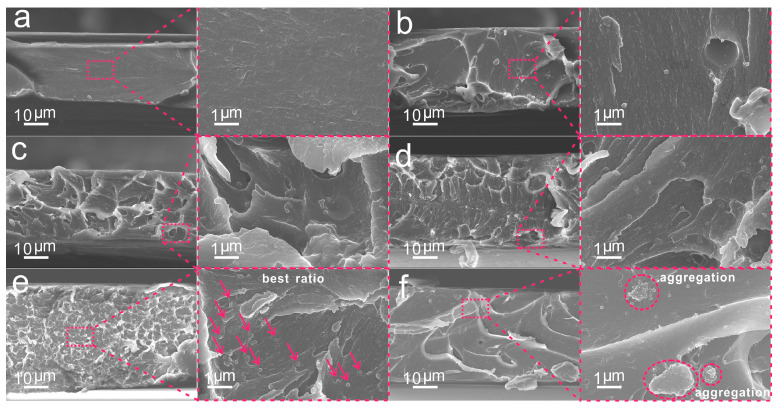
The fracture surface morphology of FPI/AMNPs films: (**a**) FPI, (**b**) FPI/AMNPs-0.1%, (**c**) FPI/AMNPs-0.3%, (**d**) FPI/AMNPs-0.5%, (**e**) FPI/AMNPs-0.7% and (**f**) FPI/AMNPs-1%. The red arrows in (**e**) represent evenly dispersed AMNPs. The red circles in (**f**) represent the aggregated AMNPs.

The fracture surface morphologies of the films were used to verify their mechanical properties (Figure 6). The fracture surface of pure FPI was flat and smooth (Figure 6a). With the addition of AMNPs, the cross-section morphology of the FPI was significantly changed. From the images of Figure 6b–e, some agglomerative grains and plastic deformed veins appeared, corresponding to the shrinkage deformation of the films. Moreover, the number of veins increased and the size decreased, and the fracture surface morphology gradually became irregular with the increasing content of AMNPs, which was attributed to the increased hydrogen bonding linkages between the FPI and AMNPs. Additionally, the entanglements within polymer chains greatly restricted the crack along the fracture surface. At this point, interface control became critical. Well-dispersed AMNPs can promote the efficient hydrogen bonding linkages with FPI to obtain a stable inorganic-organic network. At low AMNPs loading, it is difficult to find AMNPs on the fracture surface because of the pleats and the protuberances (Figure 6b–d), whereas at a higher loading (Figure 6e), homogenous veins appeared, and some AMNPs are visible on the fracture surface with uniform distribution. This can be ascribed to the induced crack because of the increases in free volume fraction at higher AMNPs contents. As the AMNPs content further increased, thick veins and pleats appeared along with obvious aggregation, as shown in Figure 6f. The high concentration of nanoparticles restricted the formation of percolated particle network and resulted in the changes in the fracture morphology. It is worth noting that the nanoparticles went through the process from volume strain to particle debonding and finally produced the shear yield. The films absorbed a lot of energy during this process and eventually reached their breaking point. However, defects generated by abundant AMNPs prevent stress dispersion, leading to fracture at the defects. In addition, a small amount of AMNPs can encourage fracture expansion and strengthen the mechanical properties of the composites.

### 2.5. Optical Properties

**Figure 7:**
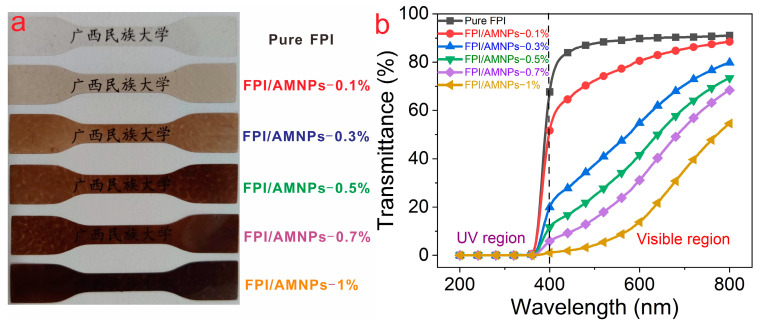
Digital photographs (**a**) and UV-vis light transmittance spectra (**b**) of FPI/AMNPs films.

A digital image of pure FPI and its composite films is displayed in Figure 7a. As can be seen, pure FPI has extraordinary transparency, allowing full visibility of the covered fonts. However, with the accumulation of AMNPs content, the color of the composite films gradually deepened with declining transparency. The results were all confirmed in the UV-vis spectra (Figure 7b). Pure FPI film exhibited the best visible light transmittance of 91.1% at 800 nm, but the transmittance of composite films in the visible light range declined as the AMNPs content increased. However, FPI/AMNPs-0.7% still possessed a good transmittance of 68.4% at 800 nm. All of the FPI/AMNPs films displayed admirable UV absorption, indicating their potential for UV-shielding applications.

### 2.6. UV-Shielding Performance

**Figure 8:**
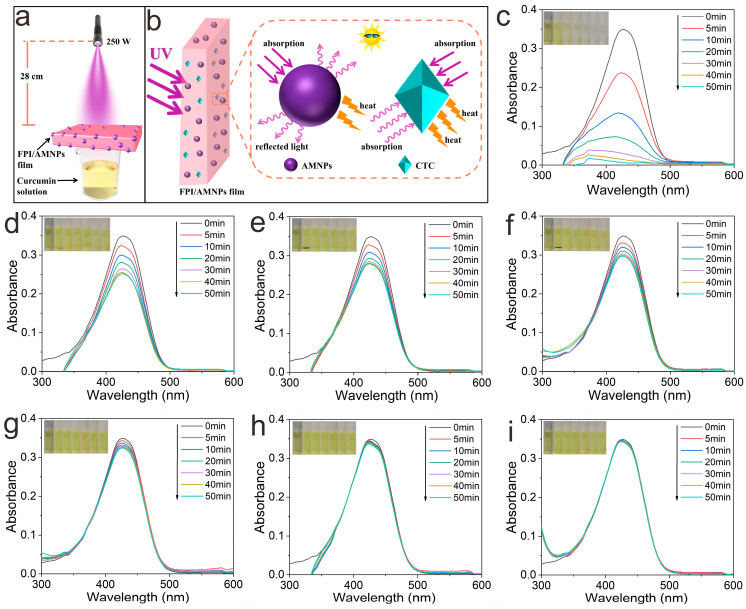
The UV-shielding measurement (**a**) and mechanism diagram (**b**) of FPI/AMNPs films. Ability of FPI/AMNPs films to block UV light: (**c**) curcumin solution’s UV visible spectrum without layer protection; (**d**–**i**) UV-vis spectra covered by FPI/AMNPs films (FPI, FPI/AMNPs-0.1%, FPI/AMNPs-0.3%, FPI/AMNPs-0.5%, FPI/AMNPs-0.7% and FPI/AMNPs-1%, respectively). The images inserted display the matching hue of the curcumin solution.

Curcumin, an acidic polyphenol, is a kind of pigment extracted from turmeric. It can be served to inhibit inflammation and has antioxidant pharmacological effects. However, it is extremely unstable under ultraviolet irradiation. The α-carbon in the structure will be decomposed into aldehydes, on which basis it will continue to be oxidized into acids [58,59,60]. As a result, curcumin is the best choice for the UV-shielding template substance to evaluate UV-shielding performance (Figure 8a). Figure 8b shows the simulation mechanism diagram of the UV-shielding experiment. In the blank control group (Figure 8c), the absorbance of curcumin decreased to 0 after 50 min of irradiation. Correspondingly, the color of curcumin changed from dark yellow to colorless, which further proved curcumin had been completely degraded. In contrast, the curcumin covered by pure FPI film was only partially degraded. The residual rate still reached 72.2% (Figure 8d), accompanied by a slight solution fade. When curcumin was covered by FPI/AMNPs films, the concentration of curcumin solution decreased less with the increase in AMNPs content (Figure 8e–i). At the same time, the color change in the curcumin solution is not gradually obvious, which is consistent with that of the initial solution.

**Figure 9:**
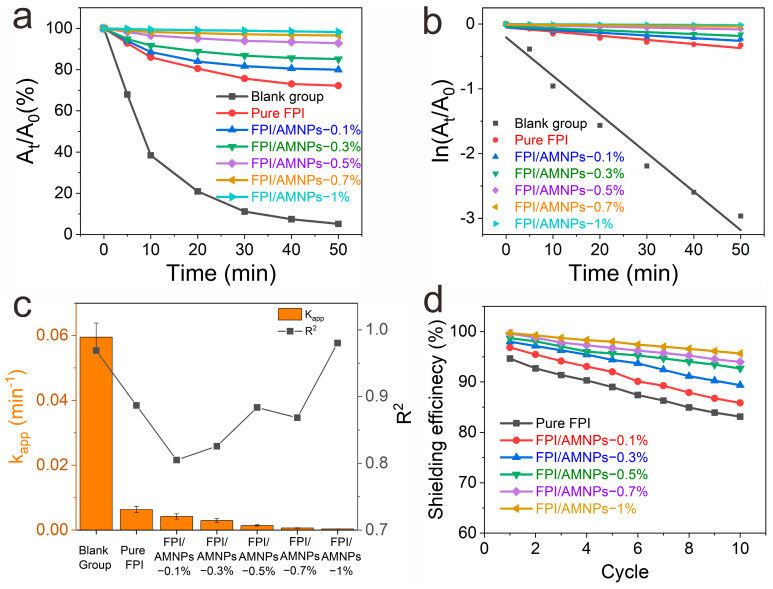
(**a**) Decay curves of the absorption intensity of the curcumin solution at 425 nm under no protection and protection by FPI/AMNPs films; (**b**) dynamic reaction rate curve of curcumin decomposition; (**c**) kinetic reaction rate constant and linear correlation coefficient curves; (**d**) repeated usability curves.

For the sake of quantifying the UV-shielding efficiency of FPI/AMNPs films, the decay and dynamic reaction rate curves of curcumin decomposition were constructed (Figure 9a,b). Figure 9a shows that curcumin decayed the fastest in the blank group. In contrast, the decay rate of curcumin shielded by pure FPI developed was dramatically reduced, indicating its shielding ability. Furthermore, the attenuation degree of curcumin decreased with the increase in AMNPs content, manifesting that AMNPs enhanced the UV-shielding effect of FPI.

The good linear relationship between ln (A_t_/A_0_) and time t further proved that the decomposition of curcumin followed the first-order linear reaction rule (Figure 9b) [61]. Among them, the largest k_app_ belonged to the blank group, while others corresponded to the curcumin covered by pure FPI, FPI/AMNPs-0.1%, FPI/AMNPs-0.3%, FPI/AMNPs-0.5%, FPI/AMNPs-0.7% and FPI/AMNPs-1%, respectively. The results showed that the UV-shielding effect of FPI/AMNPs films was proportional to the accumulation of AMNPs. The synergistic effect of FPI and AMNPs was responsible for the enhancement of UV shielding. A large amount of charge transfer complexes (CTCs) exist in or between the molecular chains of FPI, and AMNPs have excellent ultraviolet absorption. When AMNPs are uniformly dispersed in the FPI matrix, fresh CTCs will be formed between them, further enhancing the UV-shielding properties of the composite films, as shown in Figure 8b. The R^2^ value was essentially above 0.87, demonstrating a fine degree of linear fitting for this reaction (Figure 9c).

The reusability of materials is also important in the actual use process. The UV-shielding efficiency increases with the increase in AMNPs content, as displayed in Figure 9d. After 10 recycling tests, compared to pure FPI, the amplification of UV-shielding efficiency of FPI/AMNPs-1% was as high as 13%. Moreover, the UV-shielding efficiency of pure FPI was reduced by 12%, while there was only a 4% reduction in efficiency for FPI/AMNPs-1%. The above results indicated that the addition of AMNPs effectively increased the UV-shielding life of FPI. In brief, FPI/AMNPs films have excellent UV-shielding properties and reusability, and they are expected to be used as ultraviolet-shielding materials.

## 3. Experimental Section

### 3.1. Materials

4,4′-(hexafluoroisopropene) diphthalic anhydride (6FDA, 98%), 2,2′ -bis (trifluoromethyl) diaminobiphenyl (TFDB, 98%), N, N-dimethylformamide (DMF, 99.8%), N-methylpyrrolidone (NMP, 99%), sodium periodate (NaIO_4_, 98%), 1,8-dihydroxynaphthalene (1,8-DHN, 98%), acetonitrile (AR), pyridine (Py, AR), turmeric (C_21_H_20_O_6_, AR). The above reagents were purchased from Shanghai Aladdin Co., Ltd. (Shanghai, China). Acetic anhydride (AR) was acquired from Sinopharm Chemical Reagent Co., Ltd. (Shanghai, China). Before use, 6FDA was placed in 120 °C vacuum for 12 h to complete the moisture drying. DMF and NMP were purified by vacuum distillation after adding calcium hydride, and then they were stored in a 4Å molecular sieve. Other analytical grade chemicals do not require additional purification processes.

### 3.2. Preparation of AMNPs

1,8-DHN (1.60 g), acetonitrile (80 mL) and deionized water (1520 mL) were stirred in a beaker until 1,8-DHN was dissolved. An amount of 9.992 mL NaIO_4_ solution (0.5 M) was injected through a pipette gun with an additional 12 h of stirring later. The AMNPs powder was obtained by multiple centrifugations, water washing and drying at 80 °C. Subsequently, a certain amount of AMNPs were dispersed in DMF via ultrasound. After standing for 24 h, the supernatant was taken to remove large particles that could not be dispersed. The obtained AMNPs dispersion was measured to be 0.362 wt.% solid content, and it was sealed and stored in the dark.

### 3.3. Preparation of FPI/AMNPs Films

**Figure 10:**
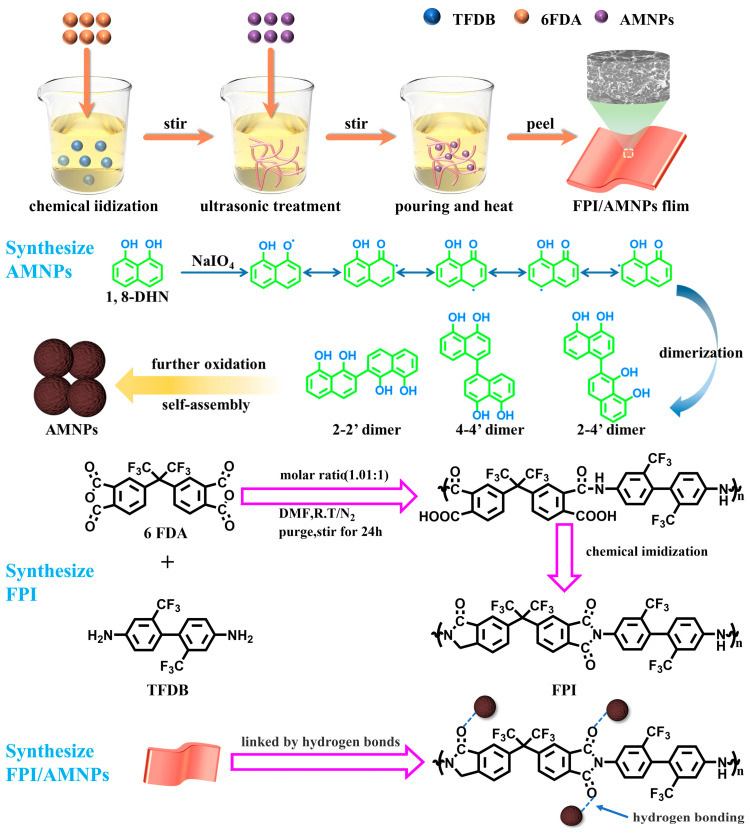
Scheme of the preparation of FPI/AMNPs films.

Schematic illustrations of the preparation of FPI/AMNPs films are displayed in Figure 10. Concretely, 3.8428 g TFDB was dissolved in 60 mL DMF. Then, 5.3827 g 6FDA was added at once with stirring again for 24 h at room temperature under N_2_ atmosphere. A mixture of 14.4 mL acetic anhydride and 7.2 mL pyridine was added bit by bit to the solution. After stirring for another 18 h, heat up to 60 °C for 6 h, 80 °C for 2 h and 100 °C for 2 h. The obtained solution was poured into excess anhydrous ethanol to obtain flocculent FPI precipitation with multiple filtration and ethanol washing before drying for 24 h at 120 °C.

In the preparation process of FPI/AMNPs film, the amount of solute (FPI) and solvent (NMP) remained constant, being set at 0.4 g and 8 mL, respectively. A certain amount of AMNPs dispersion was added, in which AMNPs content was, respectively, 0, 0.1, 0.3, 0.5, 0.7 and 1 wt.% of FPI. The mixture was evenly mixed by ultrasound and stirred for 2 h. Subsequently, the uniform mixture was poured onto a smooth glass plate with a silicone mold, and then it was transferred quickly to an oven at 60 °C until the mass of the film remained unchanged. Then, the temperature was programmed and heated at 100 °C, 150 °C, 200 °C, 250 °C and 300 °C for 1 h. The resulting FPI/AMNPs films were stripped from the mold after natural cooling, having a thickness of about 70 um, and labeled as Pure FPI, FPI/AMNPs-0.1%, FPI/AMNPs-0.3%, FPI/AMNPs-0.5%, FPI/AMNPs-0.7% and FPI/AMNPs-1% according to AMNPs content, respectively.

### 3.4. UV-Shielding Measurement

For the sake of evaluating the UV-shielding performance of FPI/AMNPs films, the degradation and recycle experiment were designed with curcumin as a template material. The detailed experimental steps are referred to in our previous work [9]. The procedures were basically the same, except that the distance between the light source and the cuvette mouth was set to 28 cm.

### 3.5. Characterization

Fourier transform infrared (FT-IR) spectra were recorded on a Nicolet IS50 spectrometer. Wide-angle X-ray diffraction (XRD) measurements were performed at room temperature on a XPERT PRO by Ni-filtered Cu-Ka (λ = 0.154 nm) radiation (40 kV, 40 mA). Field-emission scanning electron microscopy (FE-SEM) was performed on SUPRA 55 Sapphire (carl ZEISS, Jena, Germany). High-resolution transmission electron microscopy (HRTEM) was performed on JEOL JEM 2100F at an accelerating voltage of 200 kV. The mechanical properties were conducted by stress/strain test under uniaxial tension (5 mm min^−1^) using a JDL-10000N. UV-vis spectra were measured with the AOE UV-1800PC spectrometer in the transmittance mode.

## 4. Conclusions

In this article, we used 6FDA and TFDB, based on which FPI was successfully prepared. AMNPs were synthesized via oxidative oligomerization of 1,8-DHN, and a series of FPI/AMNPs nanocomposites were synthesized through hydrogen bonding between AMNPs and FPI, regulation of AMNPs content and thermal imide treatment. The results show that AMNPs (≤0.7 wt.%) were well dispersed in the FPI matrix, and nanocomposites display improved mechanical and UV-shielding properties. Adding AMNPs increases interaction and entanglement of inter-molecules of FPI, resulting in a network structure connected by hydrogen bonds. AMNPs not only change the molecular packing, which affects stress transfer, but also possess UV absorption and free radical trapping capabilities. The comprehensive effects of AMNPs on FPI show elevated tensile strength, fracture elongation and UV shielding. This work provides a facile way to prepare FPI nanocomposites with improved UV-shielding and may give guidance to the study of FPI UV-shielding materials.

## Figures and Tables

**Table 1:** Tensile test data of FPI/AMNPs films.

Sample	AMNPs (wt.%)	Tensile Strength (σ) (MPa)	Elongation at Break (*ε_max_*) (%)
FPI/AMNPs-0%	0	83.83 ± 2.01	0.90 ± 0.02
FPI/AMNPs-0.1%	0.1	117.61 ± 1.24	1.24 ± 0.03
FPI/AMNPs-0.3%	0.3	129.02 ± 1.35	1.27 ± 0.03
FPI/AMNPs-0.5%	0.5	141.28 ± 2.31	1.38 ± 0.03
FPI/AMNPs-0.7%	0.7	150.59 ± 2.45	1.40 ± 0.03
FPI/AMNPs-1%	1	130.38 ± 1.33	0.98 ± 0.02

## Data Availability

Available data are presented in the manuscript.
